# Self-Sustained Rotation of Lorentz Force-Driven Janus Systems

**DOI:** 10.1021/acs.jpcc.3c01597

**Published:** 2023-07-21

**Authors:** Gerardo Salinas, Alexander Kuhn, Serena Arnaboldi

**Affiliations:** † Université Bordeaux, CNRS, Bordeaux INP, ISM, UMR 5255, F-33607 Pessac, France; ‡ Dipartimento di Chimica, Universita degli Studi di Milano, 20133 Milano, Italy

## Abstract

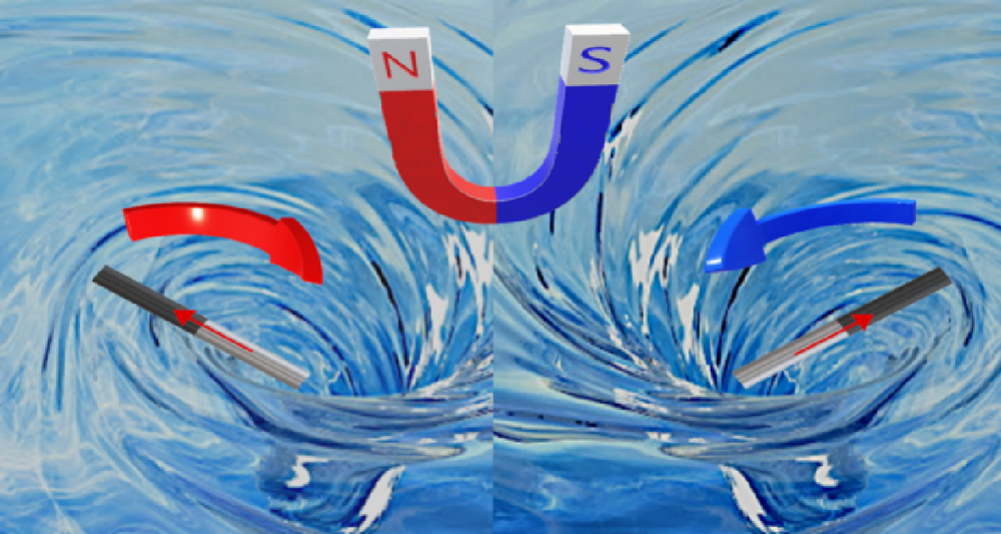

Rotation is an interesting type of motion that is currently involved in many technological applications. In this frame, different and sophisticated external stimuli to induce rotation have been developed. In this work, we have designed a simple and original self-propelled bimetallic Janus rotor powered by the synergy between a spontaneous electric and ionic current, produced by two coupled redox reactions, and a magnetic field, placed orthogonal to the surface of the device. Such a combination induces a magnetohydrodynamic vortex at each extremity of the rotor arm, which generates an overall driving force able to propel the rotor. Furthermore, the motion of the self-polarized object can be controlled by the direction of the spontaneous electric current or the orientation of the external magnetic field, resulting in a predictable clockwise or anticlockwise motion. In addition, these devices exhibit directional corkscrew-type displacement, when representing their displacement as a function of time, producing time–space specular behavior. The concept can be used to design alternative self-mixing systems for a variety of (micro)fluidic equipment.

## Introduction

Self-propelled dynamic devices have gained considerable attention due to the increasing number of applications ranging from sensing to environmental remediation.^1−6^ In this context, rotation of macro-, micro-, and nanodevices is a particular type of motion that is involved in many technological applications, *i.e*., energy transformation, pumping, or propulsion.^7−14^ Although such kind of motion can be typically triggered by connecting an object to an external motor, recently, far more sophisticated external stimuli have been proposed. Rotation of macro-, micro-, and nanodevices, powered by the Marangoni effect,^15−20^ light,^21,22^ sound,^23,24^ or electric and magnetic fields, has been achieved.^25−33^ In particular, the latter has gained considerable attention due to the possible control of the rotation trajectory (clockwise or anticlockwise), simply by changing the orientation of the magnetic field (*B*). However, this type of device requires using sophisticated experimental setups with ferromagnetic components and non-stationary magnetic flux, which limits the potential applicability.

Recently, hydrodynamic convection, produced by the induction of a local Lorentz force on the surface of a self-electrophoretic device, was efficiently used to trigger clockwise or anticlockwise motion as a function of the orientation of an external magnetic field.^34,35^ When *B* is placed orthogonal to the surface of the intrinsically polarized object, a Lorentz force is generated due to the ionic currents produced at the edges of the anodic and cathodic extremities of the endogenous bipolar device. This leads to the formation of a well-defined macromagnetohydrodynamic flux on its surface. Although this well-known phenomenon, the so-called MHD effect,^36−38^ has been extensively explored for electrodeposition,^39^ electrocatalysis,^40^ and microfluidics,^41,42^ its use for the development of more sophisticated dynamics is still a challenge. In this work, we have employed such a macro-MHD flow, produced at each extremity of a self-electrophoretic device, to design Lorentz force-driven self-propelled rotors. In this context, the thermodynamically spontaneous oxidation of a non-noble metal, magnesium (Mg) or zinc (Zn), is coupled to the kinetically more favorable reduction of protons on platinum (Pt), constituting the driving force to produce a continuous ionic current toward or away from each extremity of the rotor. Thus, when a magnetic field is oriented orthogonal to the surface of the self-polarized object, the resulting Lorentz force generates a macroscopic fluid flow, leading to the displacement of the device. Hence, the synergy between the localized hydrodynamic convection, driven by the macro-MHD vortices, and the continuous rotation of the device allows an efficient active mixing of the surrounding solution. Furthermore, since the trajectory of the rotor is directly related to the direction of the electric current and the orientation of the magnetic field, it presents specular time–space behavior.

## Methods

H_2_SO_4_ (J.T. Baker, 95–97%), sodium dodecylbenzenesulfonate (Sigma-Aldrich, technical grade), Mg foil (*d* = 0.5 mm), Zn wire (GoodFellow, 99.99%, *d* = 250 μm), H_2_PtCl_6_ H_2_O (Sigma-Aldrich, 99.9%), and blue dye solution (Crystal violet, Sigma-Aldrich) were used. All solutions were prepared with deionized water (MilliQ Direct-Q, resistivity of 18.2 MΩ·cm at 25 °C). Rotation experiments were performed in a plastic crystallizer with 10 cm diameter. A rectangular FeNdB magnet (*B* ≈ 200 mT, *A* = 98 cm^2^) was placed below the crystallizer to impose a vertical magnetic field. The Janus bimetallic rotors were obtained by dipping the required fraction of a Mg or Zn wire in a 20 mM H_2_PtCl_6_ solution, under constant stirring for 2 min, followed by ethanol washing. For the mixing experiments, the continuous pumping of the blue dye solution was carried out at a constant rate of 5 mL/h by a single-syringe pump (KDS100CE, KD Scientific). The dynamic behavior of the rotors was monitored by using a CCD camera (CANON EOS 70D, Objective Canon Macro Lens 100 mm 1:2.8). Video processing and tracking were performed with ImageJ software.

## Results and Discussion

**Scheme 1:**
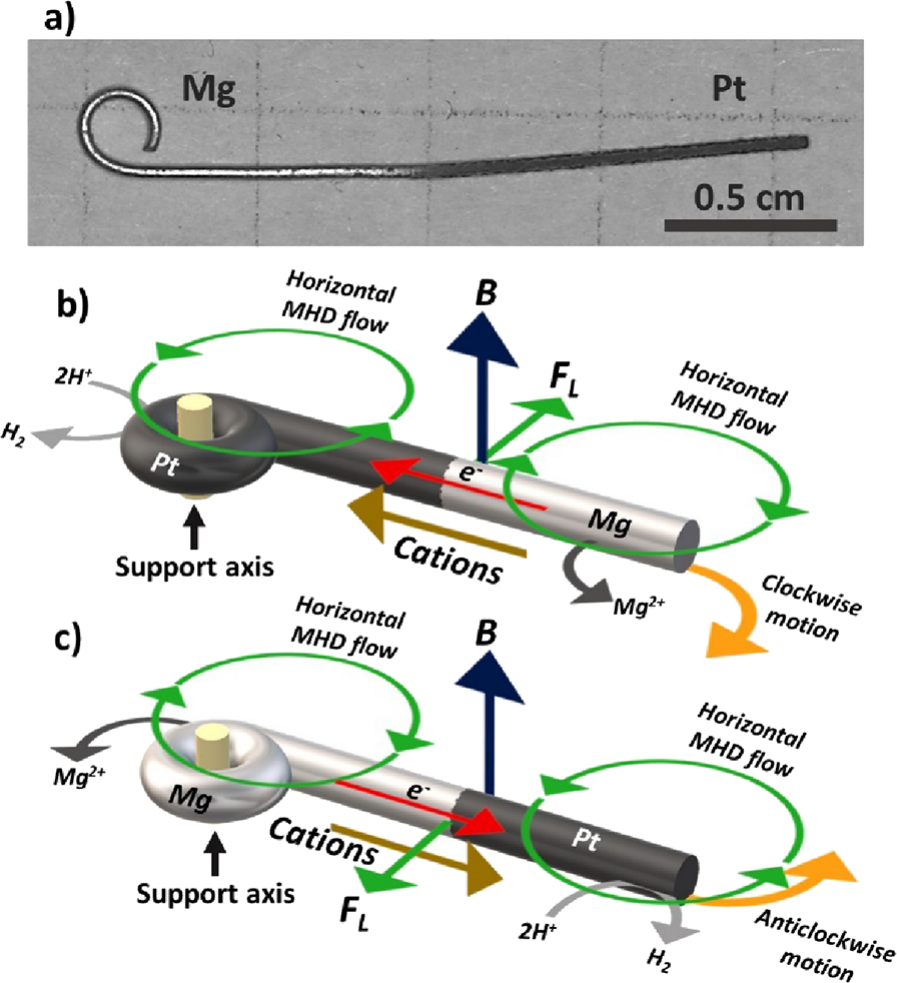
(a) Optical Picture of the Mg Rod after Asymmetric Modification in a 20 mM H_2_PtCl_6_ Solution (50% of Pt Coverage). Illustration of the Formation of the Macro-MHD Flow on the Surface of a Bimetallic Janus Rotor Where the Support Axis Is Placed on the (b) Cathodic or (c) Anodic Extremity of the Device, with a Representation of the Associated Chemical Reactions, the Spontaneous Ionic Currents, the Magnetic Field, and the Resulting Horizontal Lorentz Force

The bimetallic Janus rotors were designed by taking advantage of the spontaneous reduction of PtCl_6_^2–^ on the Mg surface (Scheme 1a). To confirm the surface modification and evaluate the morphology, scanning electron microscopy (SEM) analysis was performed with a Mg/Pt wire. As can be seen from the SEM images, the Mg extremity presents a rather homogeneous surface (Figure S1a), whereas the Pt side exhibits a porous deposit (Figure S1b). The dynamic behavior of the device was evaluated by placing the Janus rotor at the center of a Petri dish at the air/water interface of a 0.005 mM DBS/10 mM H_2_SO_4_ solution. Theoretically, in acid media, the spontaneous oxidation of Mg is coupled with the kinetically favored reduction of protons at the Pt surface. This leads to the formation of an ionic flux, away or toward the anodic and cathodic extremities, respectively. Under the influence of a magnetic field, placed orthogonal to the surface of the rotor, the resulting Lorentz force deviates the ion flux, which produces two horizontal macro-MHD flow patterns along the edges of each metallic extremity (Scheme 1b,c). Since the anode and cathode of the device are in close proximity, the sum of the two individual MHD vortices leads to an overall amplification of the local hydrodynamics, which is the main driving force of the motion (Scheme 1b,c). In addition, in recent work, it has been established that such an integrated redox system can exhibit controllable clockwise or anticlockwise rotation when it moves freely on the two-dimensional air/water interface due to an additional Lorentz force acting on the charge-compensating ion flux along the device.^34^ However, under these conditions, a possible lateral displacement toward regions where the magnetic field is no longer homogeneous has been observed. Thus, to avoid such additional motion and induce only rotation, a break of symmetry of the system is required. This was achieved by supporting the device by a thin glass tube axis, fixed at the bottom of the cell. Since in theory, the torque force that produces rotation decreases as the support axis is closer to the center of an object, we placed the support axis at the extremity of the designed rotor, that is, at the anodic (Mg) or cathodic (Pt) extremity, to favor such a dynamic behavior (Scheme 1b,c).

**Figure 1:**
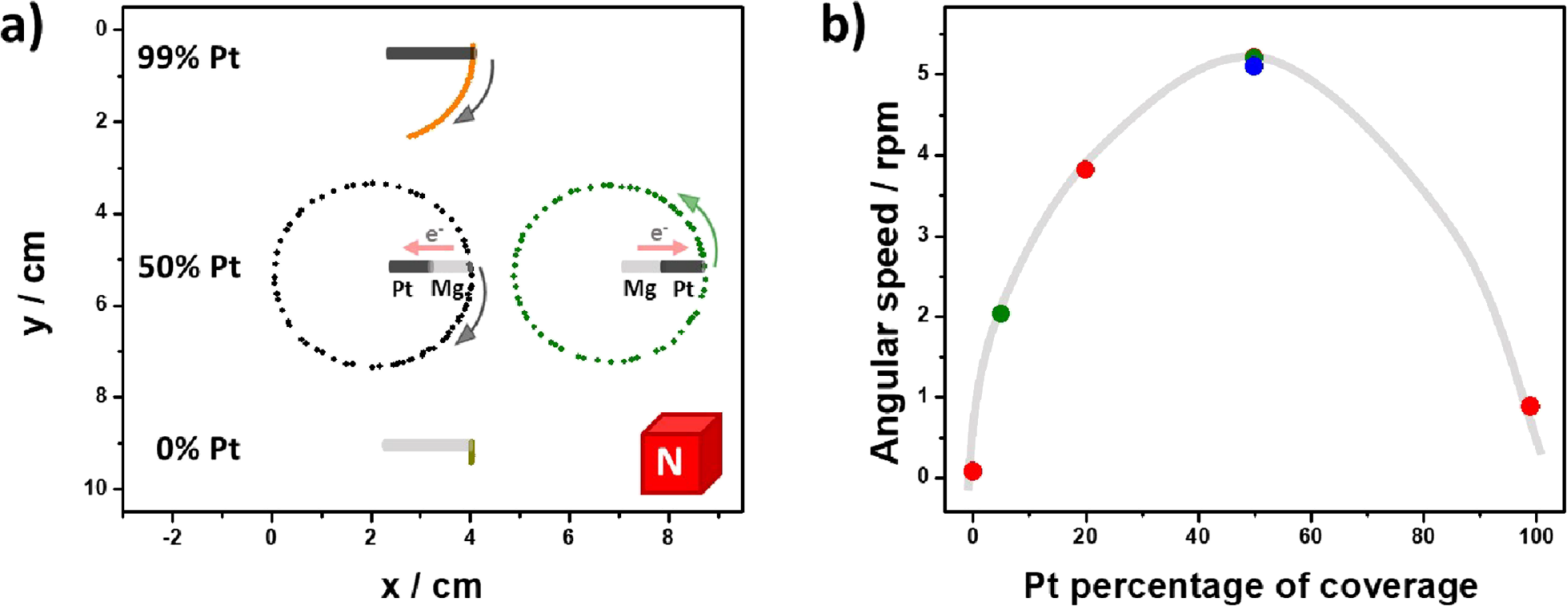
(a) Trajectory of Janus rotors with different Pt coverages (indicated in the figure) and the support axis positioned at the cathodic (black line) and anodic (green line) extremity, moving on the air/water interface (0.005 mM DBS/10 mM H_2_SO_4_) in the presence of a magnetic field (north pole up) orthogonal to the surface of the devices. (b) Rotation speed as a function of the percentage of Pt coverage with the support axis at the cathodic (red dots) and anodic (green dots) extremity. The blue dot indicates the average speed obtained with the south pole of the magnet facing upward. Global time of each experiment: 15 s.

Considering that the main driving force of the motion is associated to the overall *F*_L_, the dynamic behavior of the rotor is intimately related to the magnitude of the macro-MHD flow and the additional Lorentz force acting on the charge-compensating ion flux. It has been previously established that such an interplay is a function of the percentage of Pt that covers the Janus Mg/Pt object.^34^ When the majority of the electrons is consumed at the Mg side, due to a lack of Pt, the MHD flow dominates the motion, whereas when the catalytic reduction of protons on Pt is favored, since enough Pt is present on the device, the Lorentz force acting on the charge-compensating ion flux is dominating. To test this hypothesis, we have evaluated the influence of the composition of the device on the rotational displacement by using three independent rotors with a different Pt coverage. The devices were tested on the air/water interface of a 0.005 mM DBS/10 mM H_2_SO_4_ solution, with the support axis on the cathodic extremity. The reaction chamber was placed at the center of a rectangular FeNdB magnet (*B* ≈ 200 mT, *A* = 98 cm^2^) with the north pole facing upward. Under these conditions, all the Janus devices present a clockwise rotation, whereas only random motion is observed for the pristine Mg rotor (Figure 1a and Video S1). From the plots of the angle as a function of time, it is possible to evaluate the rotation speed (Figure S2). Rotation speed values, ranging from 0.08 to 5.3 rpm (Figure 1b, red dots), were recorded, indicating a maximum value for 50% Pt. As expected, for the device with 99% Pt coverage, rotation is limited by the relatively small magnitude of the laterally induced Lorentz force. On the other hand, the decrease in the angular speed for the rotor with a 20% Pt coverage is associated with the spatial position of the torque force along the object. In theory, the torque force that produces rotation decreases when it is located closer to the support axis. For the rotor with a 20% Pt coverage, the maximum value of *F*_L_ (and its associated torque force) is found at the interconnection region between metals, in close proximity of the support axis, which leads to a considerable decrease in angular speed.

Since the trajectory of the rotor is intimately related to the direction of the macro-MHD vortex and its concomitant overall *F*_L_, it is possible to assume that by changing the extremity where the support axis is placed, we can tune the direction of the electron flow within the object (Scheme 1). This was corroborated by evaluating the trajectory of two independent rotors, with 50 and 5% Pt, under the same experimental conditions (0.005 mM DBS/10 mM H_2_SO_4_ solution and the north pole facing upward), but placing the support axis on the anodic extremity. As expected, both devices present an anticlockwise rotation (Figure 1a, green dots and Video S1, right column) with rotation speed values around 5.2 and 2 rpm, for the devices with 50 and 5% Pt, respectively (Figure 1b, green dots). These values are in good agreement with the bell-shaped profile of the rotation speed as function of %Pt coverage. Furthermore, in addition to the trajectory control via the direction of the electron flux, clockwise and anticlockwise rotation can be inverted by changing the orientation of the magnetic field. For the same experimental conditions, the trajectory of two independent Janus rotors, with 50% Pt, and opposite position of the support axis, either on the anodic or cathodic extremity, was evaluated with the south pole of the magnetic field facing upward. Under these conditions, specular rotation was obtained, with an anticlockwise and clockwise motion for the device with the support axis at the cathodic and anodic part, respectively (Figure S3 and Video S2). In addition, rotation speed values are comparable to the ones obtained for the north pole facing upward (5 rpm) (Figure 1b, blue dot), which indicates that the dynamic behavior is not influenced by any spin polarization effect.^43−46^

**Figure 2:**
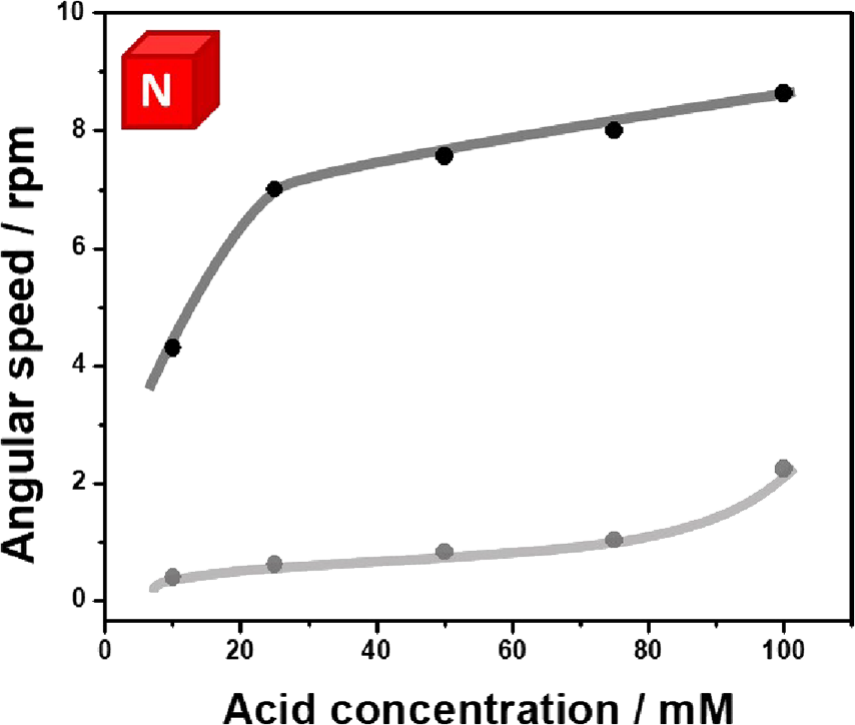
Rotation speed as a function of the H_2_SO_4_ concentration for devices with different core–shell compositions: Mg/Pt (black dots) and Zn/Pt (gray dots), moving at the air/water interface (0.005 mM DBS) in the presence of a magnetic field (north pole up) orthogonal to the surface of the devices.

After this set of experiments, where the mechanism of rotation induced by the MHD effect was demonstrated, we have studied the influence of the acid concentration on the rotation speed. In theory, an increase in the acid concentration can lead to an enhancement of the thermodynamic driving force for the anodic and cathodic reactions, which results in stronger macro-MHD vortices. In the presence of acid, when placing the north pole of the magnetic field facing upward, the Janus rotor, with 50% Pt coverage and the support axis at the anodic extremity, exhibits the characteristic anticlockwise rotation, triggered by the MHD effect (Video S3), with a linear variation of the angle as a function of time for all acid concentrations (Figure S4). Two different linear regimes for the rotation speed as a function of the H_2_SO_4_ concentration were obtained (Figure 2, black dots); below 20 mM, a steep increase in speed was observed, reaching a point where a pseudo-plateau was obtained (in the range between 20 and 100 mM). This can be associated with the formation of a MgSO_4_/MgO layer at the electrode/electrolyte interface, at concentrations above 20 mM, which partially blocks the electroactive surface.^34^ Nonetheless, such a layer does not passivate completely the electrode surface, allowing the magnetic field-enhanced self-electrophoretic propulsion mechanism to occur.

Since the spontaneous oxidation of magnesium is the driving force of the rotor, we decided to extend the same approach to alternative active metals, *e.g*., zinc (Zn). In this case, the oxidation of Zn and the reduction of protons on Pt occur spontaneously, in acid media, with a standard redox potential difference of +0.76 V, which is 3 times lower than the one produced with Mg (+2.34 V). Thus, a considerable lower rotation speed for the Zn/Pt rotors, in comparison with the Mg/Pt devices, is expected. The bimetallic Zn/Pt Janus rotors were designed following the same principle as the one used for the Mg/Pt rotors. The SEM analysis of a Zn/Pt wire confirmed the formation of a porous and granular Pt deposit on the Zn surface (Figure S5a,b). The dynamic behavior of the Zn/Pt Janus devices, with 50% Pt and the support axis positioned at the anodic extremity (Zn), was evaluated at the air/water interface of a 0.005 mM DBS solution with different H_2_SO_4_ concentrations and the north pole facing upward. As expected, all the Zn/Pt rotors exhibit an anticlockwise trajectory for all acid concentrations (Video S4). By plotting the rotation speed as a function of the H_2_SO_4_ concentration, a linear tendency was obtained in the range between 20 and 80 mM (Figure 2, gray dots). In addition, rotation speed is intimately related to the thermodynamic potential of the sacrificial anode material. This is corroborated by comparing the rotation speed of the Mg and Zn Janus rotors at 100 mM (8.4 and 2.3 rpm, respectively), where a ratio of 3.6 is obtained. Based on these results, it is possible to assume that such a Lorentz force-driven propulsion mechanism can be expanded to the coupling of alternative electrode materials. For example, copper could be an interesting candidate as a cathode instead of Pt. However, as the overpotential for hydrogen evolution on Cu is significantly higher than for Pt, the magnetohydrodynamic propulsion might be lower for such a Mg/Cu rotor.

**Figure 3:**
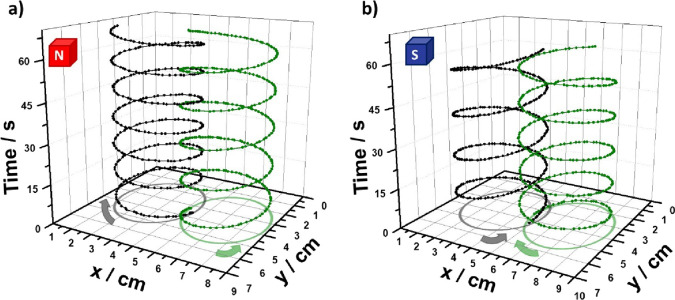
Trajectory and transient plot of a double Janus rotor system, with opposite positions of the support axis, moving at the air/water interface (0.005 mM DBS/100 mM H_2_SO_4_), as a function of the magnetic field orientation; (a) north and (b) south pole facing upward. Global time of each experiment: 1 min.

As stated above, since the trajectory of the spontaneous macroscopic mechanical rotation is intimately related to the direction of the electric current and the orientation of the magnetic field, it is possible to produce time–space specular systems. By representing the rotation as a function of time, a three-dimensional trajectory is produced, which confers the dynamic system with a specular feature. To corroborate this hypothesis, the dynamic behavior of a double-rotor setup was evaluated. At first, the two devices were placed at the air/water interface of a 0.005 mM/10 mM H_2_SO_4_ solution, with the magnetic north pole facing upward. For this set of experiments, the direction of the electric current, on each device, was controlled by placing the support axis at the cathodic or anodic extremity of the corresponding rotor. Under these conditions, a simultaneous clockwise and anticlockwise rotation, with an average speed of 5 ± 0.2 rpm, was obtained (Video S5). A random motion induced by the asymmetric formation/release of H_2_ bubbles along the object was observed in the absence of a magnetic field (Video S5). When plotting the trajectory as function of time, it is possible to visualize the formation of a clockwise and anticlockwise corkscrew-type displacement (Figure 3a), which can be considered as two time–space specular states. With the same system, the specular dynamic behavior was obtained by changing the orientation of the magnetic field (south pole facing upward), producing in this case the anticlockwise and clockwise corkscrew-type displacement, for the rotor with the support axis at the cathode and anode, respectively (Figure 3b and Video S5). Such characteristic magnetic field trajectory control resembles the rotation of light, associated with the Faraday effect. This is an optical phenomenon where a linearly polarized light, which propagates parallel to a magnetic field, rotates its polarization plane when passing through an optical transparent medium.^47^ Thus, the anticlockwise and clockwise corkscrew-type displacement, controlled by the orientation of the magnetic field, can be consider, in a first order of approximation, as a “macroscopic” version of the Faraday effect.

**Figure 4:**
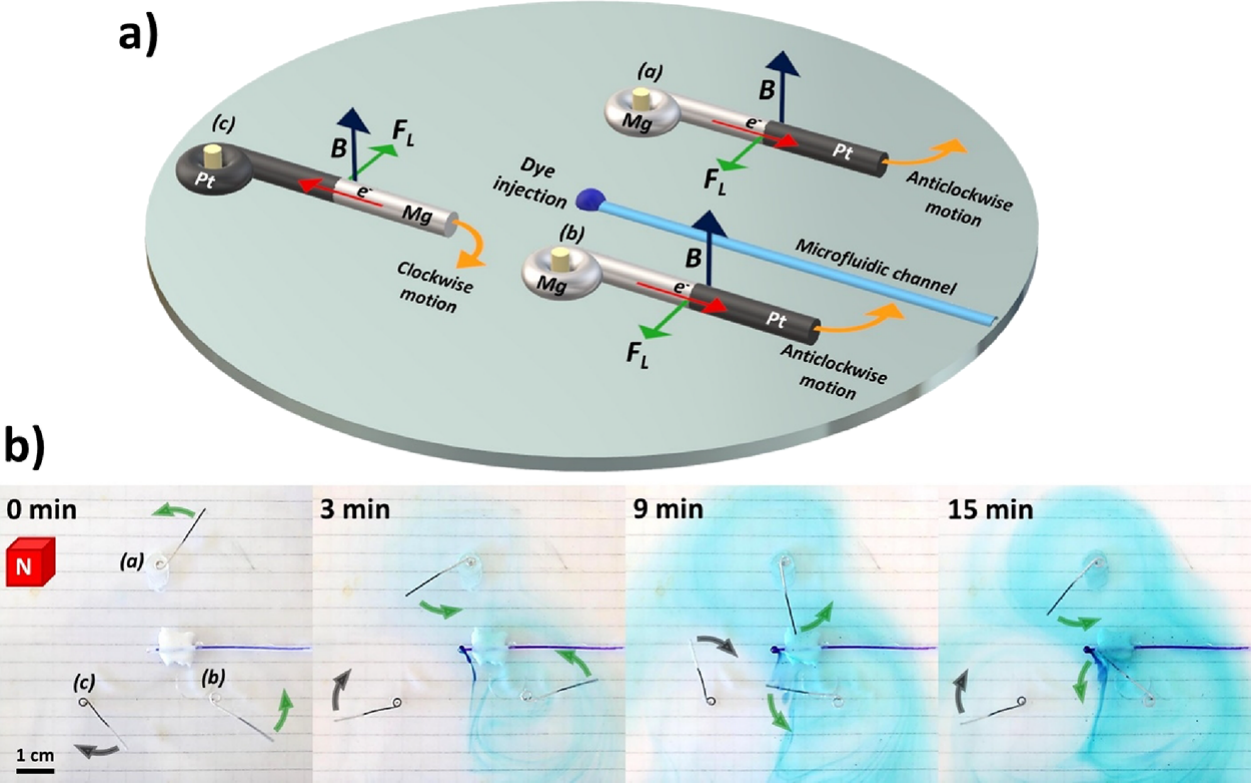
(a) Illustration of the triple rotor setup coupled to a syringe pump with a representation of the spontaneous electron flow, the magnetic field, the resulting horizontal Lorentz force, and the position of the dye injection. (b) Optical pictures at different times (indicated in the figure) of a triple Janus rotor system moving at the air/water interface (0.005 mM DBS/100 mM H_2_SO_4_) in the presence of a magnetic field (north pole up) orthogonal to the surface of the devices during the continuous pumping of a blue dye solution, illustrating the autonomous mixing capability.

Finally, since this type of rotor can be considered as a self-propelled stirrer, we tested the mixing capability of the generated dynamic behavior. To illustrate this aspect, we designed a triple Janus rotor setup coupled to a single-syringe pump, which was injecting at a constant rate (5 mL/h) a commercial blue dye (crystal violet) (Figure 4a). The three devices were placed at the air/water interface of a 0.005 mM/100 mM H_2_SO_4_ solution, with the magnetic north pole facing upward, whereas a plastic tube filled with the dye and connected to the pump was immobilized at the bottom of the solution. For reasons of simplicity, the devices are labeled as (a), (b), and (c) in Figure 4a,b. Once again, under these conditions, the trajectory is a function of the position of the support axis; thus, in such a setup, rotors (a) and (b) present an anticlockwise motion whereas (c) presents a clockwise displacement (Figure 4b and Video S6). These devices exhibit an average rotation speed of 9.5 ± 1 rpm, which remains relatively constant during the whole experiment (Figure 4b and Video S6, global time of 15 min). As it can be seen, the dynamic displacement of the rotors produces an efficient and autonomous mixing since the blue dye is spread in the solution in less than 15 min.

## Conclusions

In conclusion, we successfully designed self-propelled Janus rotors, powered by magnetohydrodynamic convection. The synergy between the spontaneous electric and ionic flux, produced by the coupled redox reactions, and the magnetic field, placed orthogonal to the surface of the device, results in an original overall driving force, able to propel the self-polarized object. An additional break of symmetry, caused by introducing a support axis placed at the anodic or cathodic extremity of the rotor, enables a controlled clockwise or anticlockwise rotation. The here proposed devices exhibit rotational displacement for relatively long periods of time until the active metal, which is the source of electrons, is completely consumed (above 60 min). In this context, it is important to point out that by fine-tuning the amount of active metal and the concentration of acid, it is possible to control the duration of the motion. Furthermore, specular trajectories can be easily generated by changing the orientation of the external magnetic field. Such a dynamic behavior is generic since alternative non-noble metals can be used for triggering directional rotation. In addition, by representing the rotation as a function of time, these devices exhibit directional corkscrew-type displacement, generating specular time–space states as a function of the direction of the electron flux and the orientation of the magnetic field. Finally, the here presented proof-of-concept provides an easy and straightforward alternative for the design of self-mixing systems, highly desired for, *e.g*., microfluidic applications. Such efficient mixing capabilities allow us to envisage the possible use of these rotating systems in electrochemical processes where mass transport, from the bulk to the electrode or *vice versa*, is one of the main constraints, *i.e*., in electroorganic synthesis or in environmental remediation. For example, due to the efficient mass transport, the self-sustained rotation could lead to higher conversion rates during zinc or magnesium-mediated reductions of carbonyl compounds^48,49^ and might be expanded to a wide variety of redox reactions with potential impact in various areas of chemistry.

## Supporting Information Available

SEM images and figures of the angle profile versus time and the trajectory of two independent Janus bimetallic rotors with the south pole facing upward (PDF)
Video S1. Rotation as a function of the Pt composition in the presence of a magnetic field (north pole up, real time) (AVI)
Video S2. Rotation of a Mg/Pt Janus rotor with the support axis at the Mg or Pt extremity, in the presence of a magnetic field (south pole up, real time) (AVI)
Video S3. Dynamic behavior of a Mg/Pt Janus rotor as a function of the H_2_SO_4_ concentration (north pole up, real time) (AVI)
Video S4. Dynamic behavior of a Zn/Pt Janus rotor as a function of the H_2_SO_4_ concentration (north pole up, 5 times accelerated) (AVI)
Video S5. Dynamic behavior of a double Mg/Pt Janus rotor system in the absence and presence of a magnetic field (5 times accelerated) (AVI)
Video S6. Mixing capability of three Mg/Pt Janus rotors (north pole, 30 times accelerated) (AVI)

## Supplementary Material

jp3c01597_si_001.pdf

jp3c01597_si_002.avi

jp3c01597_si_003.avi

jp3c01597_si_004.avi

jp3c01597_si_005.avi

jp3c01597_si_006.avi

jp3c01597_si_007.avi
