# Supplementary material for: Self-Sustained Rotation of Lorentz Force-Driven Janus Systems
Source: J Phys Chem C Nanomater Interfaces. 2023 Jul 21;127(30):14704–10. doi: 10.1021/acs.jpcc.3c01597 (PMC10405271; doi:10.1021/acs.jpcc.3c01597)
Supplement: Supplementary file 1 — jp3c01597_si_001.pdf [file jp3c01597_si_001.pdf]

Supporting Information

Self-sustained Rotation of Lorentz Force-driven Janus Systems

Gerardo Salinas[†], Alexander Kuhn[†], Serena Arnaboldi[‡]*.*

[†]Univ. Bordeaux, CNRS, Bordeaux INP, ISM, UMR 5255, F-33607 Pessac, France

[‡]Dipartimento di Chimica, Universita degli Studi di Milano, 20133 Milano, Italy

Corresponding Authors

*serena.arnaboldi@unimi.it, *kuhn@enscbp.fr

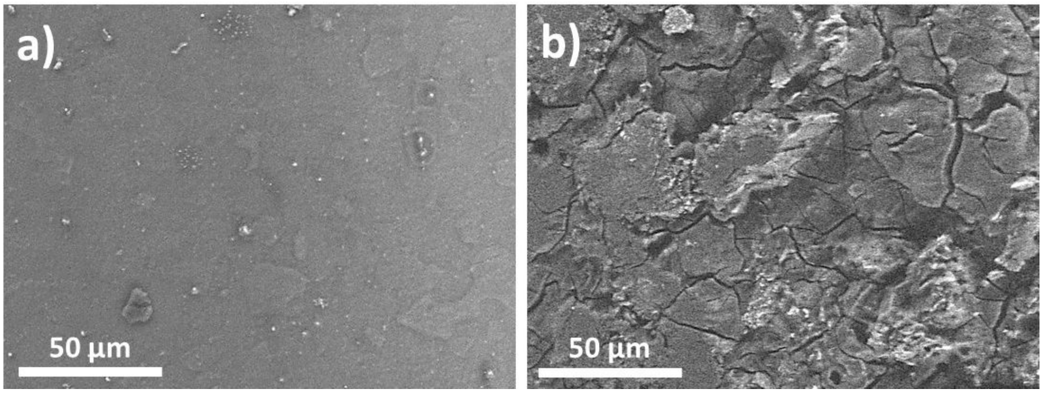

Figure S1. SEM images of the surface of (a) a pristine Mg, and (b) Pt modified Mg electrodes.

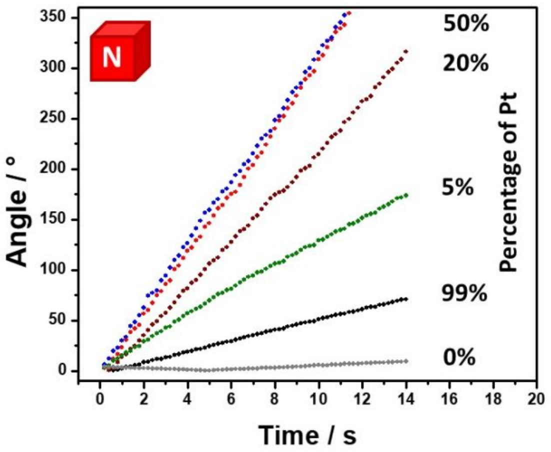

Figure S2. Plot of the angle of rotation versus time as function of the percentage of Pt coverage (indicated in the figure) obtained with different Janus rotors moving on the air/water interface (0.005 mM DBS/10 mM H_2SO_4) in the presence of a magnetic field (north pole up) orthogonal to the surface of the devices.

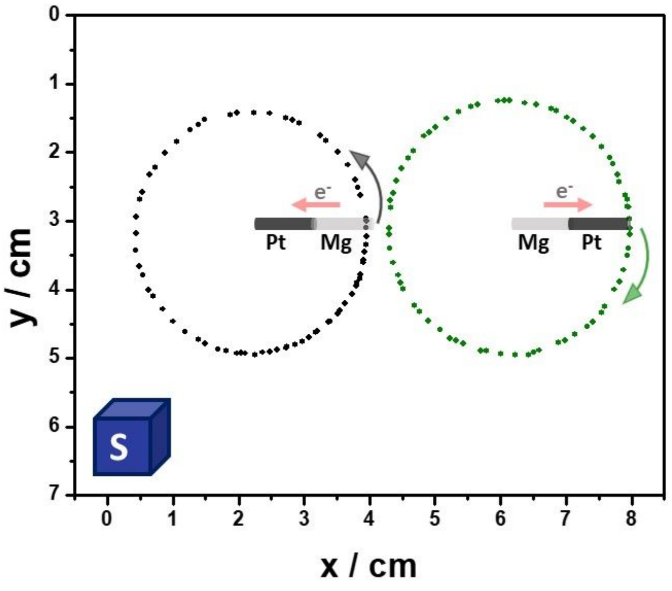

Figure S3. Trajectory of Janus rotors with two different positions of the support axis; at the platinum (black dots) and magnesium (green dots) extremity, moving on the air/water interface (0.005 mM DBS/10 mM H_2SO_4) in the presence of a magnetic field (south pole up) orthogonal to the surface of the devices.

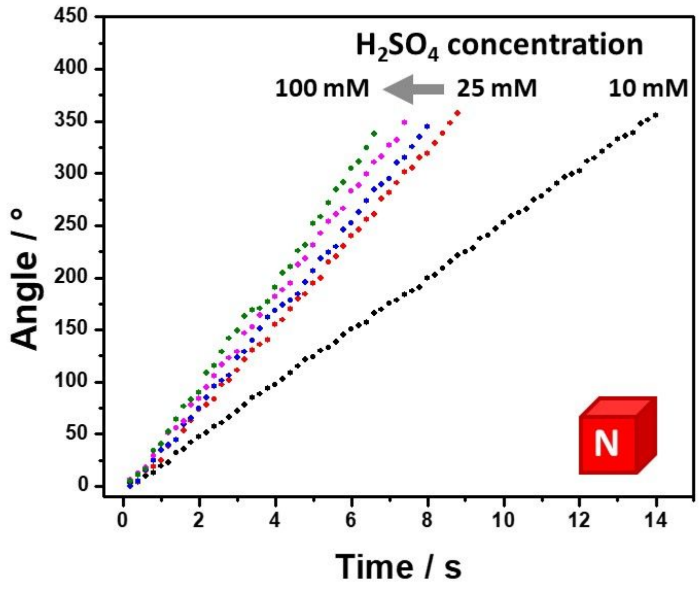

Figure S4. Plot of the angle of rotation versus time as a function of the H_2SO_4 concentration (indicated in the figure) obtained with different Janus rotors moving on the air/water interface (0.005 mM DBS) in the presence of a magnetic field (north pole up) orthogonal to the surface of the devices.

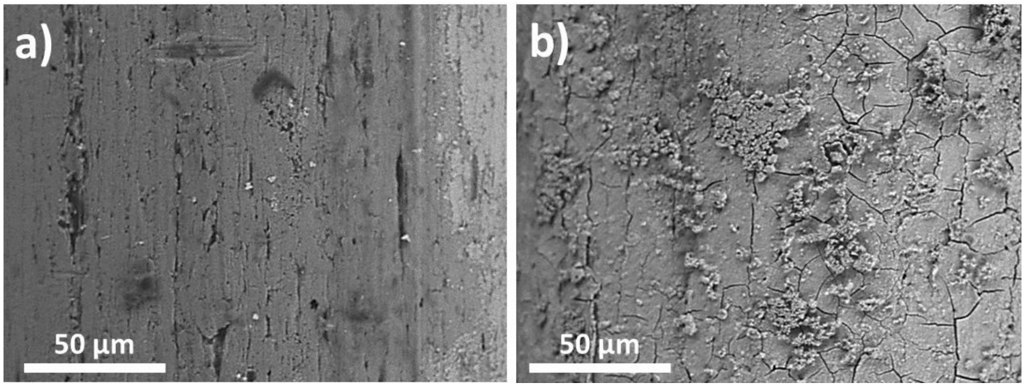

Figure S5. SEM images of the surface of (a) a pristine Zn, and (b) Pt modified Zn rotor.

Video S1. Rotation as function of the Pt composition in the presence of a magnetic field (north pole up, real time).

Video S2. Rotation of a Mg/Pt Janus rotor with the support axis at the Mg or Pt extremity, in the presence of a magnetic field (south pole up, real time).

Video S3. Dynamic behavior of a Mg/Pt Janus rotor as function of H_2SO_4 concentration (north pole up, real time).

Video S4. Dynamic behavior of a Zn/Pt Janus rotor as function of H_2SO_4 concentration (north pole up, 5x times accelerated).

Video S5. Dynamic behavior of a double Mg/Pt Janus rotor system in the absence and presence of a magnetic field (5x times accelerated).

Video S6. Mixing capability of three Mg/Pt Janus rotors (north pole, 30x times accelerated).
